# Implementation of a patient decision aid for men with localized prostate cancer: evaluation of patient outcomes and practice variation

**DOI:** 10.1186/s13012-016-0451-1

**Published:** 2016-07-02

**Authors:** Dawn Stacey, Monica Taljaard, Jennifer Smylie, Laura Boland, Rodney H. Breau, Meg Carley, Kunal Jana, Larry Peckford, Terry Blackmore, Marian Waldie, Robert Chi Wu, France Legare

**Affiliations:** 1 School of Nursing, University of Ottawa, 451 Smyth Road, Ottawa, K1H 8M5 Canada; 2 Clinical Epidemiology Program, Ottawa Hospital Research Institute, 1053 Carling Avenue, Ottawa, ON K1Y 4E9 Canada; 3 Ages Cancer Assessment Clinic, The Ottawa Hospital, 501 Smyth Road, Ottawa, ON K1H 8L6 Canada; 4 Population Health, Faculty of Health Sciences, University of Ottawa, 451 Smyth Road, Ottawa, ON K1H 8M5 Canada; 5 Department of Surgery, Division of Urology, University of Ottawa, 501 Smyth Rd, Ottawa, K1H 8L6 Canada; 6 Department of Surgery, Division of Urology, 537-750 Spadina Cr. E., Saskatoon, S7K 3H3 Canada; 7 Prostate Cancer Canada Network, Ottawa, Canada; 8 Quality and Continuous Improvement, Acute and Emergency Services Branch, Saskatchewan Ministry of Health, Regina, Canada; 9 Postgraduate Medical Education, University of Ottawa, Ottawa, Canada; 10 Research Centre CHU de Quebec-Universite Laval, Quebec, Canada

**Keywords:** Implementation science, Patient decision aid, Prostate cancer, Comparative case studies, Study proposal

## Abstract

**Background:**

Men with localized prostate cancer often have unrealistic expectations. Practitioners are poor judges of men’s preferences, contributing to preference misdiagnosis and unwarranted practice variation. Patient decision aids (PtDAs) can support men with decisions about localized prostate cancer. This is a comparative case study of two strategies for implementing PtDAs in clinical pathways for men with localized prostate cancer, evaluating (a) PtDA use; (b) impact on men, practitioners, and health system outcomes; and (c) factors influencing sustained use.

**Methods/design:**

Guided by the Knowledge to Action Framework, this comparative case study will be conducted using administrative data, interviews, and surveys. Cases will be bound by geographic location (one hospital in Ontario; province of Saskatchewan) and time. Eligible participants will be all men newly diagnosed with localized prostate cancer, with outcomes assessed using administrative data and interviews. Nurses, urologists, radiation oncologists, and managers will be surveyed and a smaller sample interviewed. Cases will be established for each setting with findings compared across cases. Changes in the proportions of men given the PtDA over 2 years will be determined from administrative data. Factors associated with receiving the PtDA will be explored using multivariable logistic regression analysis. To assess the impact of the PtDA, outcomes will be described using mean and standard deviation (men’s decisional conflict) and frequency and proportions (practitioners consulted, uptake of treatment). To estimate the effect of the PtDA on these outcomes, adjusted mean differences and odds ratios will be calculated using exploratory multivariable general linear regression and binary or multinomial logistic regression. Factors influencing sustained PtDA use will be assessed using descriptive analysis of survey findings and thematic analysis of interview transcripts.

**Discussion:**

Determining how to embed PtDAs effectively within clinical pathways for men with localized prostate cancer is essential. PtDAs have the potential to strengthen men’s active role in making prostate cancer decisions, enhance uptake of shared decision-making by practitioners, and reduce practice variation. Our team of researchers and knowledge users will use findings to improve current PtDA use and consider scaling-up implementation.

**Electronic supplementary material:**

The online version of this article (doi:10.1186/s13012-016-0451-1) contains supplementary material, which is available to authorized users.

## Background

Although patient decision aids (PtDAs) are effective interventions for translating evidence for patients [1], they are not routinely used in clinical practice [2]. Our study aims to close the gap between what is currently known about PtDAs from research studies and what is done with PtDAs when they are implemented in routine clinical practice. Men with localized prostate cancer face a difficult decision because there are four main options (e.g., surgery, external radiation, brachytherapy, and active surveillance) with different potential benefits and harms. Given little evidence to indicate that one option is better than another, the chosen option should be consistent with men’s informed preferences based on weighing benefits and harms across options [3–5].

When asked to take an active role in making these difficult decisions, patients often experience decisional conflict and have unrealistic expectations [6–9]. Decisional conflict is “personal uncertainty about which course of action to take when choice among competing options involves risk, regret, or challenge to personal life values” [7–9]. Adults with unresolved decisional conflict are more likely to delay decisions, feel regret, be dissatisfied, and blame doctors for bad outcomes [10, 11]. Moreover, without effective decision support, patients may be exposed to more costly options without any better outcomes [12, 13]. Studies in Canada and the USA found that urologists and radiation oncologists often provide unbalanced information on options in favour of their own expertise, and they are not able to correctly guess men’s preferences [14, 15]. Practice variations in age-standardized rates of surgery for prostate cancer are 32 to 57 % across Ontario, Canada [16]. This variation may be unwarranted given that the best option depends on men’s preferences [17].

For adults facing difficult decisions, providing a structured approach to decision-making such as PtDAs helps empower individuals, resolves decisional conflict, and reduces unwarranted practice variation [13, 17]. PtDAs are booklets and/or videos that provide balanced information on options (benefits/harms), help clarify patients’ preferences, and guide patients making decisions with their practitioner [18]. A systematic review of 115 trials of PtDAs (including prostate cancer treatment) found that patients exposed to PtDAs are more involved in decision-making with improved knowledge, more realistic expectations of outcomes, and enhanced agreement between options chosen and patients’ values [13, 19]. Despite strong evidence, few PtDAs are used in clinical practice [2]. In 17 implementation studies, factors interfering with their use were healthcare professionals having inadequate training, being indifferent about using them, lacking confidence in their content, and being concerned about disrupting workflows [2]. Only one implementation study in England involved men with prostate cancer [20]. These men had improved knowledge and values-choice agreement, shifted rates from surgery to radiation, and rated the PtDA positively. Staff said the PtDA was used by men at home and off-loaded work in busy clinics. Other studies have shown that few practitioners attempt to involve patients in decision-making and fewer adjust care to patients’ preferences [21], thus leading to a “silent misdiagnosis of clients’ preferences,” a key determinant of health system performance as a whole [22]. Hence, it is important to study implementation of PtDAs to understand approaches that increase sustained use and have positive impacts on patient, practitioner, and health system outcomes.

In our previous research with 192 patients in an ambulatory oncology program, only half were offered treatment choices and those offered choices were more likely to have an active role in decision-making [23]. Patients whose preferred role was different from their actual role preferred more involvement. Next, we explored using PtDAs for men with localized prostate cancer [24]. A team of researchers and knowledge users appraised PtDAs against the International Patient Decision Aid Standards [25] to identify two higher quality PtDAs (one was booklet only; one was booklet plus video). Interviews with prostate cancer survivors, urologists, oncologists, and nurses rated the PtDAs positively for plain language, helpful information, and ability to share with family [24]. Men wanted more information on sexual effects and brachytherapy. Factors perceived to influence their use were men’s preferred level of involvement, staff time to distribute, practitioners’ own agenda, and practitioner/manager buy-in. Since September 2010, the higher quality PtDA with booklet and video from Health Dialog was integrated into the clinical pathway for men diagnosed with localized prostate cancer at The Ottawa Hospital. In 2010, a key health goal of Saskatchewan was to implement a shared decision-making framework to engage patients in decisions about their treatment options [26]. In May 2013, the prostate cancer surgical pathway in Saskatchewan started using PtDAs with men having localized prostate cancer [27]. Implementation has not been evaluated in either program and anecdotally, not all men receive a PtDA.

Researchers on this team have also updated the Cochrane review of PtDAs [13], the Cochrane review of interventions for uptake of shared decision-making [28], and reviews on shared decision-making training [29–31], PtDA implementation [2], and PtDA used with decision coaching [32, 33]. None of the studies measured sustained the use of PtDAs or factors influencing sustained use. A recent study of PtDA implementation for patients with cystic fibrosis in Canada revealed 85 and 92 % PtDA usage at 1 and 2 years, respectively, but findings were based on nurse reporting rather than more objective administrative data [34].

The overall aim of this comparative case study is to evaluate two strategies for implementing PtDAs in clinical pathways for men with localized prostate cancer by measuring (a) the use of PtDA over a 2-year period after implementation; (b) impact of the PtDA on men, practitioners, and health system outcomes; and (c) factors influencing sustained use (or not) for each strategy.

## Methods/design

Our comparative case study will use mixed methods and will be guided by the Knowledge to Action Framework [35, 36]. Following Yin’s approach, the cases will be bound by location (prostate cancer clinical pathway at one hospital in one provincial health system and the prostate cancer surgical pathway in another province) and time period (2 years after PtDA implementation) [37]. The Knowledge to Action framework focuses on specific implementation factors essential for success in real world, naturalistic environments [35, 36]. The Action Cycle was activated by a problem identified by the knowledge users (e.g., men want to be more involved in prostate cancer decisions), and the knowledge solution is a PtDA. Then, we will identify interventions to overcome identified barriers. This proposed implementation study is focused on the final phases of the Framework: monitor use, evaluate outcomes, and measure sustained use. Sustainability requires monitoring use for 2 or more years [38]. We obtained ethics approval at The Ottawa Health Science Network Research Ethics Board (#20150604-01H), the Saskatoon Health Region Research Ethics Board (#BEH-15-382), Reginal Qu’Appelle Health Region Research Ethics Board (REG-15-125), and the University of Saskatchewan Behavioural Research Ethics Board (BEH#15-382).

The *setting* for our study is prostate cancer healthcare services that implemented PtDAs in the clinical pathways: The Ottawa Hospital in Ontario (in 2010) and the Ministry of Health in the Province of Saskatchewan (in 2013). These prostate cancer healthcare services have urologists and radiation oncologists serving populations of about 1 million each. Annually, about 400 men have consultations for prostate cancer at The Ottawa Hospital Cancer Assessment Clinic and about 600 in Saskatchewan. Within the clinical pathway, men diagnosed with localized prostate cancer are asked to use the PtDA at home in preparation for the consultation. Nurses, trained in interprofessional shared decision-making [31], discuss men’s questions prior to the physician consultation.

*Participants* for the interviews are men who have received a diagnosis of localized prostate cancer (including their partners) and received services at The Ottawa Hospital or in Saskatchewan. Other stakeholder participants who have varying levels of influence in the prostate cancer programs include urologists, radiation oncologists, nurses, and managers.

*Procedures* focus on collecting clinical administrative data (clinical databases, electronic/paper-based health records) and conducting a survey and interviews (Table 1). Routinely collected administrative data sources will be reviewed to identify men newly diagnosed with localized prostate cancer for calendar years of 2011–2012 in Ottawa and 2014–2015 in Saskatchewan. Data will be retrieved on (a) their age, co-morbidities with severity, prostate cancer characteristics; (b) practitioners’ documentation of the decision-making consultation; (c) uptake of treatment or surveillance; (d) whether given the PtDA or not; and (e) level of decisional conflict (Table 2).

**Table 1:** Proposed study data collection elements and analysis

Outcome	Participants	Data source	Analysis
Proportion given the PtDA	Men with localized prostate cancer	Clinical administrative^a^ data (~*n* = 1000)	Proportion with 95 % confidence intervals for each case study
Men’s outcomes: decisional conflict	Men with localized prostate cancer	Clinical administrative^a^ data (~*n* = 1000)	Linear regression (decisional conflict) to compare those who were given the PtDA to those who were not
Practitioner’s outcome: factors influencing PtDA use	Urologists, radiation oncologists, and nurses in prostate programs	Mailed survey (~*n* = 40)	Multivariable ordinal logistic regression analysis of practitioner’s reported use of PtDA with factors reported in the survey as covariates. Subgroup analysis will be done by types of practitioner (e.g., urologists, radiation oncologists, nurses)
Health system outcomes: uptake of treatment (including surveillance) and practitioner consultations	All men with localized prostate cancer	Clinical administrative^a^ data (~*n* = 1000)	Multinomial and binary logistic regression analysis of treatment uptake and practitioner consultations with receipt of the PtDA as main predictor, and adjusting for patient demographic and clinical characteristics.
Factors influencing PtDA implementation	Key stakeholders in each program	Individual interviews (~*n* = 30–40)	Thematic analysis guided by the Knowledge to Action Framework

^a^Clinical administrative data for all men for 2-year period after implementing the PtDA

**Table 2:**
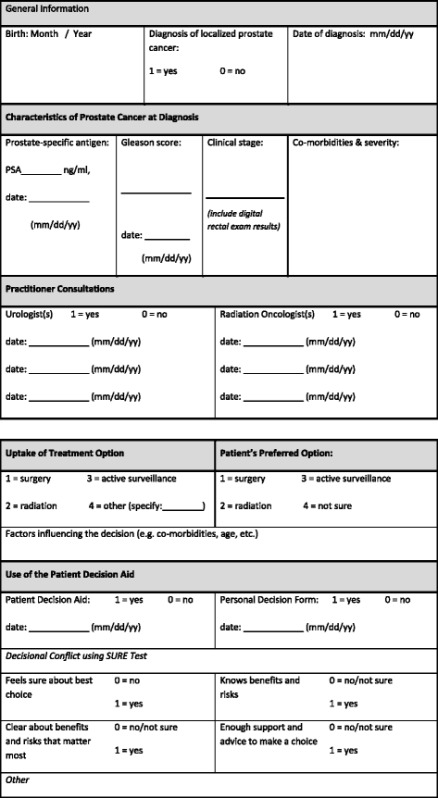
Data collection tool: prostate cancer PtDA implementation study

A survey will be sent to urologists (*n* = 8–10) per site, radiation oncologists (*n* = 5-7), and nurses (*n* = 4–6) in the prostate programs to assess their use of PtDA and factors influencing use. Eligible participants will receive a mailed package that will include a cover letter with the study purpose and information on how the information will be used and the survey with a stamped pre-addressed envelope. To enhance response rates, Dillman’s approach will be used with reminders at 2, 4, and 5 weeks [39].

Interviews will be conducted with a purposeful sample of men given/not given the PtDA (and/or their partners), nurses, managers, radiation oncologists, and urologists in each program (Table 1). Men newly diagnosed with prostate cancer during the last 3 years post-PtDA implementation will be contacted by a healthcare professional from the circle of care or through the local prostate cancer support groups. Purposeful sampling will be used to identify other stakeholders having varying levels of influence in the prostate cancer programs (e.g., nurses, urologists, radiation oncologists, and managers). Prospective participants will be invited to participate in an interview by (a) announcement of the study at staff meetings, (b) hard copy posters on staff bulletin boards, and (c) email to staff (Ottawa only). A letter explaining the study will be provided to prospective participants, and written informed consent will be obtained (Additional files 1 and 2). For healthcare practitioners, a semi-structured interview guide developed using the Knowledge to Action Framework [35] will be used to explore perceptions of PtDAs, factors influencing PtDA sustained use, interventions required to overcome remaining barriers, and concurrent initiatives that may have influenced PtDA use. For men given or not given a PtDA, a semi-structured interview guide was developed to learn more about their experience with how the decision was made to treat or monitor their prostate cancer. All participants will be asked to complete demographic questions.

*Interventions* will be identified to address barriers interfering with the use of PtDAs if the prevalence of use is found to be less than 80 %. If PtDA use is 80 % or higher, strategies for monitoring sustained use with feedback mechanisms will be established [40]. Successful implementation of evidence in clinical practice requires tailored interventions based on identified barriers [41].

*Instruments* to be used have been found to be valid, reliable, and have been previously been used in PtDA studies. The four-item SURE test is used to screen for decisional conflict to inform clinical practice [42–44]. It has moderate reliability (Cronbach Alpha 0.65) and discriminates between those who had/had not made a choice. Factors influencing PtDA use by practitioners will be measured using the 12-item Continuing Professional Development Reaction Questionnaire (Cronbach Alpha 0.77-0.85) [45]. Potential environmental barriers influencing PtDA use will be taken from a Barriers Survey [46].

*Outcomes* are based on the Knowledge to Action Framework [35]. After knowledge is implemented (e.g., PtDA), its uptake should be monitored to determine how and to what extent it is used [35]. We plan to use administrative data to measure the proportion of men who were given the PtDA and qualitative interviews to explore men’s use. The next phase of the Framework is to measure the impact of knowledge use on outcomes specific to patients, providers, and healthcare systems [35]. We will measure the impact of PtDA use on men’s decisional conflict using administrative data; practitioners’ and patients’ consultations using qualitative interviews; and impact on healthcare systems using administrative data to measure uptake of treatment and types of practitioners consulted during decision-making. Measuring impact of PtDA on these outcomes will ensure that PtDA use is influencing quality indicators as observed in randomized controlled trials without any unintended consequences [13, 40]. Sustaining the use of the knowledge is the last phase in the Action Cycle of this Framework [35, 47]. Given that barriers can change with PtDA use [46], we will assess barriers using a practitioner survey and qualitative interviews.

### Analysis

Two cases will be developed with a rich description of the context and outcomes with comparisons within and across the cases [37]. For PtDA use, the proportion of men given the PtDA will be calculated for each site with 95 % confidence intervals (Table 1). We anticipate about 600 men diagnosed with localized prostate cancer over 2 years at The Ottawa Hospital Cancer Assessment Clinic and 400 in the Provincial Prostate Pathway in Saskatchewan. Using conservative estimates for proportions given the PtDA of 50 and 90 % in Ottawa and Saskatchewan, respectively, these sample sizes are adequate to estimate the true proportions given the PtDA with margins of error +4.0 % in Ottawa and +2.9 % in Saskatchewan using two-sided 95 % confidence intervals. Characteristics and outcomes of patients who did and did not receive the PtDA will be described at each site using mean and standard deviation for continuous variables, and frequencies and proportions for categorical variables.

Factors associated with receiving the PtDA will be explored at each site and overall using multivariable logistic regression analysis by entering patient characteristics (e.g., age, co-morbidity, severity of disease, cancer characteristics) as well as provider characteristics (e.g., sex, years of experience, discipline). To explore differences in the effect of these characteristics across the two sites, site and interactions between the characteristics and site will be included in the model. If the proportion using the PtDA is 50 % in Ottawa and 90 % in Saskatchewan, according to a commonly used rule of 10 events per independent variable, the maximum number of variables that can be included in these analyses is 66; thus, we have more than the required degrees of freedom available to fit our models. The extent of missing data will be tabulated, and characteristics of those with and without missing data will be compared. If more than 5 % missing data is observed among predictors, the use of multiple imputation will be explored. The analysis will account for clustering by provider through the inclusion of random intercepts for each provider. The model will be estimated using pseudo-likelihood or maximum likelihood in SAS v9.3.

Outcomes between those given the PtDA and those not given it will be described at each site using mean and standard deviation (decisional conflict) and frequency and proportions (uptake of treatment, type of practitioner consulted). To estimate the effect of the PtDA on these outcomes after accounting for potential confounders, we will conduct exploratory multivariable general linear regression and binary or multinomial logistic regression at each site and overall. The analysis will adjust for patient characteristics associated with the use of the PtDA, and patient clinical and demographic characteristics potentially associated with the outcomes. To explore differences between the sites, we will include site and its interaction with the receipt of the PtDA into the model. Results will be expressed as adjusted mean differences and adjusted odds ratios together with 95 % confidence intervals.

For impact on practitioner outcomes and sustained use, we will identify remaining barriers influencing practitioner PtDA use with the survey and interview data. Audio-taped interviews will be transcribed verbatim and analyzed qualitatively by two team members using thematic analysis. We expect to reach saturation by 12 to 15 interviews in each case study [48].

The study *Timeline* is 12 months: 3 months for start-up; 5 months for collecting clinical administrative data, conducting the survey, and interviewing; 2 months for data cleaning/analysis; and 2 months for drafting case studies and disseminating findings.

### Dissemination plan

The knowledge users on the research team will disseminate results within their organizations and through their networks. As well, we plan to publish the results in open access peer-reviewed journals and provide presentations at relevant international and national oncology and implementation science conferences. We plan to disseminate a brief policy report and our research tools (in English/French) through our networks and on research websites (decisionaid.ohri.ca; www.ktcanada.ohri.ca; decision.chaire.fmed.ulaval.ca). This end of grant knowledge translation plan has the potential to improve ongoing use of PtDA for men with localized prostate cancer, highlight the impact on various outcomes, and inform approaches for implementing PtDAs in other programs.

## Discussion

Our deliverables are findings on the actual use of PtDAs for men with localized prostate cancer using two different implementation approaches in two different healthcare systems over a 2-year period. We will measure outcomes on patients, practitioners, and the healthcare system to ensure findings are consistent with benefits of PtDAs reported in randomized controlled trials of PtDAs without unintended consequences. Importantly, we will also learn about strategies required to ensure sustained use of PtDAs. In summary, deliverables include an approach for implementing PtDAs, ways to monitor their use to provide feedback to knowledge users, and strategies required to support their sustained use.

Our study results will advance knowledge about implementation of PtDA into clinical practice. These findings can be used by knowledge users on our team for making decisions about improving implementation of PtDAs and scaling up their use with patients having prostate cancer or other cancers. We anticipate that the implementation of PtDAs enhanced patient-centred care by increasing patient involvement in health decisions, thereby boosting public confidence in healthcare services [20] and improving quality of life [49] to advance the Canadian Cancer Society’s mission. Health policy documents in Saskatchewan [50], Ontario [51], and in other countries (e.g., USA, UK, Australia, Germany) call for a “patient first” approach to healthcare that can be improved by patients using PtDAs and making decisions with their practitioner, thus, creating demand for evidence on sustainable PtDA implementation approaches that our study will provide.

### Research team

Fundamental to our study is an integrated knowledge translation approach [52] whereby knowledge users representing policy makers, practitioners, and a patient are collaborating as research team members. Knowledge users helped define the research objectives and provided critical feedback on our proposed study [53]. Engaging knowledge users in meaningful ways during the proposal development should maximize the likelihood of producing findings of use to them and enhance uptake of our study findings [54]. Knowledge users will be engaged in interpreting study findings and disseminating results.

Our proposed study will be managed from the Ottawa Hospital Research Institute: a multidisciplinary research-intensive environment with knowledge translation research as one of its strategic priorities. Our research team is internationally recognized as a leader in PtDA’s development, evaluation, and implementation. We have a highly productive team of researchers, knowledge users, and trainees with expertise in oncology, knowledge translation, health services research, and health policy. Our team is in a supportive research environment, has established collaborations with necessary expertise to conduct the proposed study, and can transfer findings to inform health policy and services.

## Abbreviations

PtDA, patient decision aid

## Additional files

Additional file 1: Participant consent form. (DOCX 91 kb)

Additional file 2: Participant informed consent form. (DOC 186 kb)
