# Supplementary material for: Implementation of a patient decision aid for men with localized prostate cancer: evaluation of patient outcomes and practice variation
Source: Implement Sci. 2016 Jul 2;11:87. doi: 10.1186/s13012-016-0451-1 (PMC4930601; doi:10.1186/s13012-016-0451-1)
Supplement: Supplementary file 1 — Participant consent form. (DOCX 91 kb) [file 13012_2016_451_MOESM1_ESM.docx]

**Project Title:** Implementation of a prostate cancer decision aid within routine clinical practice Study

**Researcher(s):** Dr. Kunal Jana, MD, FRCSC, Clinical Associate Professor, Department of Surgery, Division of Urology, University of Saskatchewan, Phone: 306-653-3255

**Purpose(s) and Objective(s) of the Research:**

- Men diagnosed with localized prostate cancer often have 3 main options: surgery, radiation, or being followed without treatment. Patient decision aids (DVD/booklets) are designed to help patients in making decisions and learn about their options. Although the prostate cancer program started using patient decision aids, not all men are given the patient decision aid and/or use them. The goal is to study how patient decision aids are used for men with localized prostate cancer and identify factors that make it harder or easier to use patient decision aids as part of the process of care. This study is taking place at two sites across Canada: The Ottawa Hospital and the Province of Saskatchewan. We estimate that 65 participants will be enrolled in the study with half from The Ottawa Hospital and half from Saskatchewan
- You are being asked to participate in this research study because within the last two years:
  - you were told you have prostate cancer OR
  - you are the partner or spouse of someone who was told they have prostate cancer OR
  - you are a urologist, radiation oncologist, nurse, or manager/administrator who is involved in delivery of health services to men with prostate cancer

**Procedures:**

- The study involves individual interviews with men with prostate cancer who used a patient decision aid, men with prostate cancer who did not use a patient decision aid, their partners (spouses), nurses, urologists, radiation oncologists, and managers/administrators.
- You will be asked to participate in one interview at a convenient time and location. The interview will take about 20 to 30 minutes. You may skip any questions that make you uncomfortable or that you do not wish to answer. After the research team has begun the analysis of all the interviews, we may contact you in a short telephone call to review the findings and make sure it fits with your view. Interviews will be audio recorded. You may choose not to be recorded and still participate.
- For health care professionals, the study will be done on employee’s time.
- Please feel free to ask any questions regarding the procedures and goals of the study or your role.

**Funded by:** Canadian Cancer Society

**Potential Risks:**

- There are no known or anticipated risks to you by participating in this research.
- You might find some of the questions uncomfortable. You do not have to answer any questions that make you uncomfortable.
- There is a risk of loss of confidentiality of your data, but the researchers have taken precautions to protect your privacy and the risk is considered very small.

**Potential Benefits:**

- You may not receive any direct benefit from participating in this study. Your participation may help the research team understand how to best support men with localized prostate cancer who are making decisions about treatment with their doctor. This may benefit future patients and/or healthcare professionals.

**Compensation:**

- You will not be paid for participating in this study.

**Confidentiality:**

- All information collected during your participation in this study will be identified with a unique study number, and will not contain information that identifies you, such as your name, address, etc. The link between your unique study number and your name and contact information will be stored securely and separate from your study records, and will not leave the study site.
- Any documents leaving the hospital will contain only your unique study number. This includes publications or presentations resulting from this study. Information that identifies you will be released only if it is required by law.
- **Storage of Data:**
  - Data will be stored in a locked filing cabinet in a locked office by Dr. Kunal Jana.
  - Research records will be kept for 10 years, after this time they will be destroyed, where all paper records will be shredded and all electronic records will be securely deleted.

**Right to Withdraw:**

- Your participation is voluntary and you can answer only those questions that you are comfortable with. You may withdraw from the research project for any reason, at any time without explanation or penalty of any sort.
- *[for patients/spouses]* Whether you choose to participate or not will have no effect on the medical care, education, or other services to which you are entitled or are presently receiving at this institution.
- *[for health care professionals]* Whether you choose to participate or not will have no effect on your position, employment, or how you will be treated.
- Should you wish to withdraw, the study team will no longer continue the interview for research purposes. You may choose to have the previously collected data withdrawn from the study.
- Your right to withdraw data from the study will apply until results have been disseminated. After this date, it is possible that some form of research dissemination will have already occurred and it may not be possible to withdraw your data.

**Follow up:**

- To obtain results from the study, please contact Dr. Kunal Jana 306-653-3255

**Questions or Concerns:**

- Contact the researcher using the information at the top of page 1;
- This research project has been approved on ethical grounds by the University of Saskatchewan Research Ethics Board (REB) and Regina Qu'Appelle Health Region REB. Any questions regarding your rights as a participant may be addressed to that committee through the Research Ethics Office [ethics.office@usask.ca](mailto:ethics.office@usask.ca) (306) 966-2975. Out of town participants may call toll free (888) 966-2975.

**Consent**

Your signature below indicates that you have read and understand the description provided; I have had an opportunity to ask questions and my/our questions have been answered. I consent to participate in the research project. A copy of this Consent Form has been given to me for my records.

|  |  |  |  |  |
| --- | --- | --- | --- | --- |
| *Name of Participant* |  | *Signature* |  | *Date* |

______________________________ _______________________

*Researcher’s Signature Date*

By initialing below, I acknowledge that I have given permission for my interview to be audio recorded for transcription purposes

|  |
| --- |
| *Initials of Participant* |

***A copy of this consent will be left with you, and a copy will be taken by the researcher.***
