# Supplementary material for: Implementation of a patient decision aid for men with localized prostate cancer: evaluation of patient outcomes and practice variation
Source: Implement Sci. 2016 Jul 2;11:87. doi: 10.1186/s13012-016-0451-1 (PMC4930601; doi:10.1186/s13012-016-0451-1)
Supplement: Supplementary file 2 — Participant informed consent form. (DOC 186 kb) [file 13012_2016_451_MOESM2_ESM.doc]

***
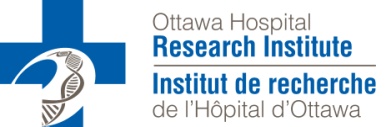

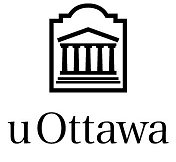


***

**PARTICIPANT INFORMED CONSENT FORM**

**Title of Study: Implementation of a prostate cancer decision aid within routine clinical practice Study**

**Local Site Principal Investigator (PI): Dawn Stacey (613-737-8899 ext 73817)**

**Funding Agency**: Canadian Cancer Society

Participation in this study is voluntary. Please read this Participant Informed Consent Form carefully before you decide if you would like to participate. Ask the study investigator and study team as many questions as you like.

**Why am I being given this form?**

You are being asked to participate in this research study because within the last two years:

- - you were told you have prostate cancer OR
  - you are the partner or spouse of someone who was told they have prostate cancer OR
  - you are a urologist, radiation oncologist, nurse, or manager/administrator who is involved in delivery of health services to men with prostate cancer

**Why is this study being done?**

Men diagnosed with localized prostate cancer often have 3 main options: surgery, radiation, or being followed without treatment. Patient decision aids (DVD/booklets) are designed to help patients in making decisions and learn about their options. Although the prostate cancer program started using patient decision aids, not all men are given the patient decision aid and/or use them. The goal is to study how patient decision aids are used for men with localized prostate cancer and identify factors that make it harder or easier to use patient decision aids as part of the process of care. This study is taking place at three sites in Canada: The Ottawa Hospital and St.Paul’s Hospital in Saskatoon and Pasqua Hospital in Regina, Saskatchewan.

We estimate that 65 participants will be enrolled in this study with half from The Ottawa Hospital and half from Saskatchewan.

**How is the study designed?**

The study involves individual interviews with men with prostate cancer who used a patient decision aid, men with prostate cancer who did not use a patient decision aid, their partners (spouses), nurses, urologists, radiation oncologists, and managers/administrators.

**What is expected of me?**

You will be asked to participate in one interview at a convenient time and location. The interview will take about 20 to 30 minutes. You may skip any questions that make you uncomfortable or that you do not wish to answer. After the research team has begun the analysis of all the interviews, we may contact you in a short telephone call to review the findings and make sure it fits with your view. Interviews will be audio recorded. You may choose not to be recorded and still participate. You have the choice at the end of this form.

**How long will I be involved in the study?** The entire study will last one year. Your participation will be a one-time interview lasting about 20-30 minutes and you may be contacted for a short telephone call following the analysis. For health care professionals, the study will be done on employee’s time.

**What are the potential risks I may experience?** You might find some of the questions uncomfortable. You do not have to answer any questions that make you uncomfortable.

**Can I expect to benefit from participating in this research study?** You may not receive any direct benefit from participating in this study. Your participation may help the research team understand how to best support men with localized prostate cancer who are making decisions about treatment with their doctor. This may benefit future patients and/or healthcare professionals.

**Do I have to participate? What alternatives do I have? If I agree now, can I change my mind and withdraw later?**

[*for patients/spouses*] Your participation in this study is voluntary. The alternative to this study is not to participate.

You may decide not to be in this study, or to be in the study now, and then change your mind later without affecting the medical care, education, or other services to which you are entitled or are presently receiving at this institution. If you withdraw your consent, the study team will no longer collect your personal health information for research purposes. Information given to the sponsor before you cancel this consent may still be used.

[*for health care professionals*] Your participation in this study is voluntary. The alternative to this study is not to participate. You may decide not to be in this study, or to be in the study now, and then change your mind later. Your decision will not affect your current or future employment at the Ottawa Hospital.

If you withdraw your consent, the study team will no longer collect your personal identifying information for research purposes. You may choose to have the previously collected data withdrawn from the study.

**Will I be paid for my participation or will there be any additional costs to me?**

No, you will not be paid for participating in this study.

**How is my personal information being protected?**

- All information collected during your participation in this study will be identified with a unique study number, and will not contain information that identifies you, such as your name, address, etc.
- The link between your unique study number and your name and contact information will be stored securely and separate from your study records, and will not leave this site.
- Any documents leaving the Ottawa Hospital will contain only your unique study number. This includes publications or presentations resulting from this study.
- Information that identifies you will be released only if it is required by law.
- For audit purposes only, your original study records may be reviewed under the supervision of Dr. Dawn Stacey’s staff by representatives from:
  - the Ottawa Health Science Network Research Ethics Board (OHSN-REB),
  - the Ottawa Hospital Research Institute
  - Research records will be kept for 10 years, after this time they will be destroyed, where all paper records will be shredded and all electronic records will be securely deleted.

**Who do I contact if I have any further questions?**

If you have any questions about this study, please contact Dr. Dawn Stacey at 613-737-8899 ext. 73817. The Ottawa Health Science Network Research Ethics Board (OHSN-REB) has reviewed this protocol. The Board considers the ethical aspects of all research studies involving human participants at the Ottawa Hospital. If you have any questions about your rights as a study participant, you may contact the Chairperson at 613-798-5555, extension 16719.

***
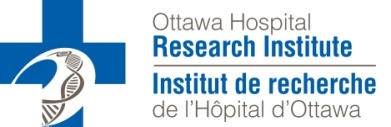

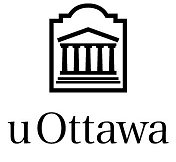


***

**Implementation of a prostate cancer decision aid within routine clinical practice Study**

**Consent to Participate in Research**

- I understand that I am being asked to participate in a research study about study about how patient decision aids are used for men with localized prostate cancer.
- This study was explained to me by ___________________________.
- I have read, or have had it read to me, each page of this Participant Informed Consent Form.
- All of my questions have been answered to my satisfaction.
- If I decide later that I would like to withdraw my participation and/or consent from the study, I can do so at any time.
- I voluntarily agree to participate in this study.
- I will be given a copy of this signed Participant Informed Consent Form.

I agree to be audio taped. Yes  No  Initials ___

_____________________________ ____________________________ _________________

Participant’s Printed Name Participant’s Signature Date

**Investigator or Delegate Statement**

I have carefully explained the study to the study participant. To the best of my knowledge, the participant understands the nature, demands, risks and benefits involved in taking part in this study.

_____________________________ ____________________________ _________________

Investigator/Delegate’s Printed Name Investigator/Delegate’s Signature Date

Was the participant assisted during the consent process? ❑ Yes ❑ No

❑ The consent form was read to the participant/substitute decision-maker, and the person signing below attests that the study was accurately explained to, and apparently understood by, and consent was freely given by the participant/substitute decision-maker.

❑ The person signing below acted as a translator for the participant/substitute decision-maker during the consent process. He/she attests that they have accurately translated the information for the participant/substitute decision-maker, and believe that the participant/substitute decision-maker has understood the information translated.

__________________________ __________________________ ________________

Name of Person Assisting (Print) Signature Date
